# ﻿Reinstatement of the independent specific status of *Camellia angustifolia*, a tea plant (Camellia sect. Thea, Theaceae) from Guangxi, China

**DOI:** 10.3897/phytokeys.267.174664

**Published:** 2025-11-28

**Authors:** Huiqun Deng, Xianjun Liao, Xiangqin Yu, Zhusheng Liu, Shixiong Yang

**Affiliations:** 1 Guangxi Key Laboratory of Tea Plant Germplasm Innovation and Resource Utilization, Guangxi Research Institute of Tea Science, Guilin 541004 Guangxi, China Guangxi Research Institute of Tea Science Guilin China; 2 Guilin Field Scientific Observation and Research Station for Crop Germplasm Resources, Ministry of Agriculture and Rural Affairs, Guilin 541004 Guangxi, China Ministry of Agriculture and Rural Affairs Guilin China; 3 Guangxi Field Scientific Observation and Research Station for Tea Resources, Guilin 541004 Guangxi, China Guangxi Field Scientific Observation and Research Station for Tea Resources Guilin China; 4 CAS Key Laboratory for Plant Diversity and Biogeography of East Asia, Kunming Institute of Botany, Chinese Academy of Sciences, Kunming 650201, Yunnan, China Kunming Institute of Botany, Chinese Academy of Sciences Kunming China

**Keywords:** *Camellia sinensis* var. *pubilimba*, morphology, taxonomic treatment, Theaceae

## Abstract

*Camellia angustifolia* Hung T. Chang was published in 1981 and was treated as a synonym of C. sinensis var. pubilimba in 1992. Field investigation and herbarium research have revealed remarkable morphological differences between *C. angustifolia* and C. sinensis var. pubilimba. Evidence from morphology—particularly the variations in indumentum on young branches, leaves, and flowers, the size of the sepals, and the thickness of the pericarp—suggests that *C. angustifolia* should not be reduced to C. sinensis var. pubilimba. We hereby reinstate the independent specific status of *C. angustifolia*. A detailed description, including the flower morphology of *C. angustifolia*, is provided. In addition, the incorrect information regarding the type specimen in the protologue of *C. angustifolia* has been rectified.

## ﻿Introduction

*Camellia angustifolia* Hung T. Chang was described as a new member of the Camellia L. sect. Thea Griffith, based on a fruit specimen collected from Dayaoshan County (current Jinxiu County), Guangxi, China (Chang 1981a). In the protologue, the author stated that the species was similar to *C. sinensis* (L.) Kuntze but differed by its branchlets and leaves glabrous, leaves narrow lanceolate, pericarp thick (4–5 mm) and sepals large (6–9 mm long) (Chang 1981a). Ming (1992) pointed out that the large sepals of *C. angustifolia* did not represent a significant difference from *C. sinensis*, because sepal enlargement during the fruiting period was not uncommon in the latter. Therefore, he reduced *C. angustifolia* to the synonymy of C. sinensis var. pubilimba Hung T. Chang.

Since the 1990s, we have been searching for *C. angustifolia* in its type locality and the surrounding areas. We had sporadically collected a few specimens that were suspected to be of this species outside its type locality in the early days, but it was not until 2021 that we finally confirmed the discovery of this plant in its type locality. Subsequently, additional specimens of this plant at various developmental stages (from flower bud, open flower, and young fruit to mature fruit) were collected from a wider range of locations. After careful scrutiny of the literature, specimens (including type material), and living plants, it is concluded that *C. angustifolia* is remarkably different from C. sinensis var. pubilimba in morphology and should be reinstated as an independent species.

## ﻿Materials and methods

Relevant specimens of C. sect. Thea, including the type material of *C. angustifolia* and C. sinensis var. pubilimba, conserved at herbaria IBK, IBSC, KUN, PE, and SYS (acronyms based on Thiers (2025, continuously updated)), and taxonomic literature were checked. Field collections and observations were also conducted on the living plants of these two taxa.

## ﻿Results and discussion

### ﻿The valid publication

*Camellia angustifolia* was published as a new species in 1981 by the same author in two publications (Chang 1981a: 96 and 1981b: 119), with the same type. Chang (1981a) was published in February 1981, earlier than Chang (1981b) (April 1981), so it is the valid publication (Art. 6 Note 2 of the ICN).

### ﻿The type specimens

**Figure 1.**
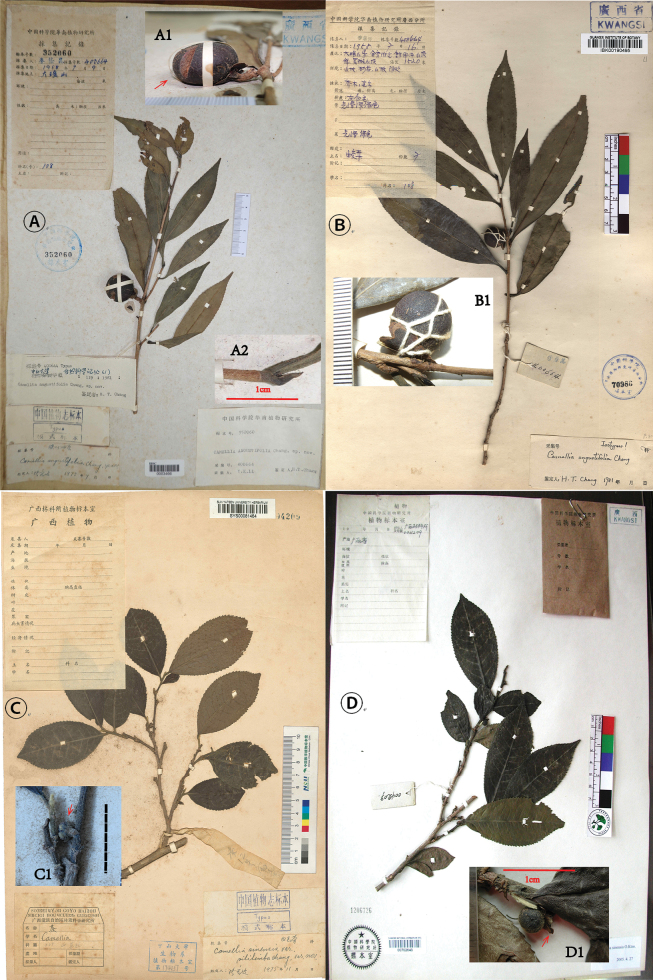
Type specimens. **A.** Holotype of *C. angustifolia* (IBSC); **A1.** Showing the thick pericarp (arrow: the thinnest part); **A2.** showing the glabrous branchlet, petiole, and the terminal bud with sparse pubescence; **B.** Isotype of *C. angustifolia* (IBK); **B1.** Showing the shape of the fruit and the large glabrous sepals; **C.** Holotype of C. sinensis var. pubilimba (SYS); **C1.** Showing the hairy terminal bud, branchlet, and petiole, as well as the small, abaxially hairy sepals (arrow: sepals); **D.** Isotype of C. sinensis var. pubilimba (PE); **D1.** Showing the obvious indumentum on the terminal bud, branchlet, petiole, abaxial leaf surface, and the adaxial surface of the sepals (arrow: sepals). **A.** Photographed by Yunfei Deng; **B, D.** Photographed by Shixiong Yang; **C.** Photographed by Qiang Fang and Lijuan Luo.

In the protologue of *C. angustifolia*, a single collection, Y.K. Li 400644 deposited in IBSC, was designated as the type (Chang 1981a). Two sheets of this collection were found in IBSC and IBK, respectively. However, both of them are definitely not *C. angustifolia* but *Platycodon grandiflorus* (Jacq.) A. DC., a member of the family Campanulaceae. After careful research, it was confirmed that Chang (1981a, b) provided an incorrect specimen number, which was followed by Chang and Bartholomew (1984) and Ming (1992, 2000). A nearby collection, Y.K. Li 400664, is actually the type of *C. angustifolia*. We found two sheets of this collection in IBSC and IBK, respectively. On the sheet in IBSC (IBSC0003466, holotype), there is a nomenclature label “*Camellia angustifolia* Chang, sp. nov.” handwritten by Zhang Hongda (Hung T. Chang) in September 1975, and a “Typus” stamp (Fig. 1A). On the sheet in IBK (IBK00190495, isotype), there is a label “isotypus of *C. angustifolia*,” which should have been written by T.L. Ming based on the handwriting (Fig. 1B).

It is worth noting that there are differences in collection information between the holotype and the isotype. The collection record of the isotype provides comprehensive collection information, including the collector, collection number, collection date (16 July 1958), collection locality (Dayaoshan County as well as detailed sublocations), habitat, altitude, life form, and morphological characteristics of leaves and fruits, as well as uses, etc. (Fig. 1B). However, on the collection record sheet of the holotype, in addition to providing the same collector and collection number as the isotype, only the collection date (9 July 1958) and locality (Dayaoshan), which differ from those of the isotype, were noted (Fig. 1A). By checking the collector’s record book (Yinkun Li), which is stored in IBK, it was finally confirmed that the collection information attached to the isotype is correct.

The holotype (not in good condition) and an isotype of C. sinensis var. pubilimba were traced from SYS (SYS00081464) and PE (PE00702640), respectively, and were collected in Lingyun, Guangxi, China (Fig. 1C, D). We also collected substantial specimens of this taxon from its type locality and the surrounding area.

### ﻿Morphological analysis

**Figure 2.**
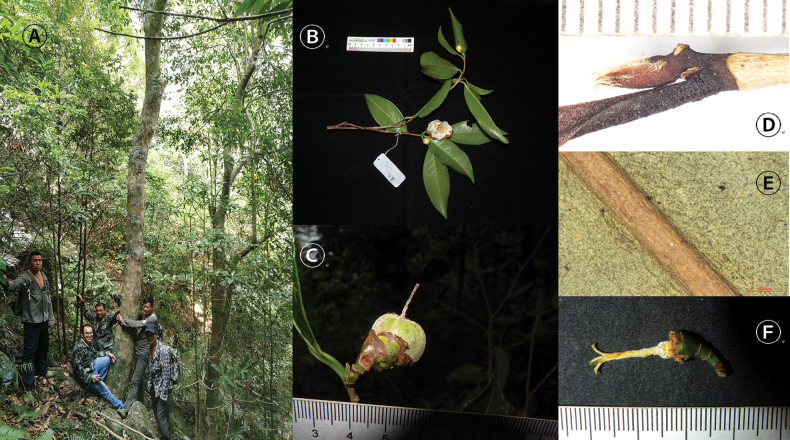
*Camellia angustifolia* Hung T. Chang. **A.** Habit; **B.** Flowering branch. **C.** Young fruit with sepals; **D.** Young branch with terminal bud and petiole; **E.** The back of a leaf; **F.** Pistil. **A, B, D.** From S.X. Yang *&* P.M. Ye 6755 (KUN); **C.** From S.X. Yang *&* P.M. Ye 6756 (KUN); **E, F.** From S.X. Yang *&* P.M. Ye 6757 (KUN). Photographed by Shixiong Yang.

**Figure 3.**
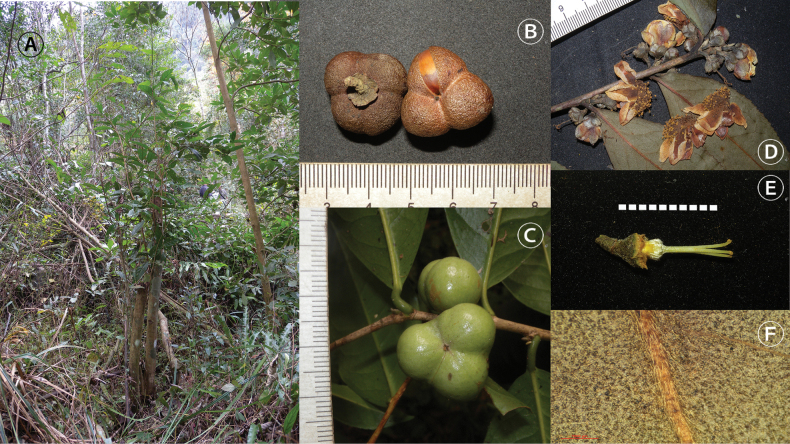
Camellia sinensis var. pubilimba Hung T. Chang. **A.** Habit; **B, C.** Fruit; **D.** Flowering branch; **E.** Pistil; **F.** The back of a leaf. **A–C.** From S.X. Yang 5528 (KUN); **D–F.** From S.X. Yang 6556 (KUN). Photographed by Shixiong Yang.

All specimens, including the types and living plants in the wild, reveal that *C. angustifolia* is remarkably distinct from C. sinensis var. pubilimba, as evidenced by an array of morphological characters (Table 1 and Figs 1–3). The main differences are as follows.

**Table 1.** Morphological comparison between *Camellia angustifolia* and C. sinensis var. pubilimba.

**Character**	*C. angustifolia*	*C. sinensis* **var.** * pubilimba *
Life form	arbor, 10–20 m tall	shrub, 1–3 (–5) m tall
Terminal buds	sparsely to densely pubescent	densely pubescent
Branchlet	glabrous	pubescent
Abaxial surface of leaf blade	glabrous	pubescent
Diameter of flower	4–5 cm	2.5–3.5 cm
Pedicel	6–12 mm long, glabrous	5–10 mm long, pubescent
Size of sepals	5–9 mm	2–4 mm
Indumentum of sepals	outside glabrous, inside sericeous	outside pubescent, inside sericeous
Size of petals	1.5–3 × 1.5–2 cm	1.5–2 × 1.2–2 cm
Indumentum of petals	glabrous on both surfaces	outside pubescent
Ovary	3-loculed, densely tomentum	3-loculed, densely tomentum
Style	1.6–2 cm long, pubescent almost the entire length	ca. 1 cm long, glabrous or base pubescent
Shape of fruit	globose	triangular oblate
Size of fruit	3–4 cm in diam.	2–3 cm in diam.
Thickness of pericarp	2–3 mm	≤1 mm

Firstly, C. sinensis var. pubilimba is primarily diagnosed by the presence of obvious indumentum on the young branches, the back of the leaves, the petioles, the pedicels, and the abaxial surfaces of the perianths (sepals and petals) (Chang 1981a, b) (Figs 1C, C1, D, D1, 3D, F). In contrast, the corresponding parts of *C. angustifolia* are glabrous (Figs 1A, A2, B, B2, 2C, D, E), which was seriously overlooked when Ming (1992) reduced *C. angustifolia* to C. sinensis var. pubilimba.

Secondly, the sepals of *C. angustifolia* (Figs 1B1, 2C) are significantly larger than those of C. sinensis var. pubilimba (Figs 1C1, D1, 3D) (5–9 mm long vs. 2–4 mm). This constitutes a stable difference between the two taxa and contradicts the view of Ming (1992).

Thirdly, the fruits of *C. angustifolia* are globose (Figs 1A, B, 2C), whereas those of C. sinensis var. pubilimba are usually triangular oblate (Fig. 3C). What is particularly noteworthy is that the pericarp of *C. angustifolia* is thicker than that of C. sinensis var. pubilimba. The description “pericarpio 4–5 mm crasso” in the protologue of *C. angustifolia* (Chang 1981a) was not entirely accurate. This refers to the thickness of the pericarp around the fruit tip, where it is the thickest (Fig. 1A1). In fact, the thinnest part of the pericarp is only 2–3 mm thick (as indicated by the arrow in Fig. 1A1), but it is still obviously thicker than that of C. sinensis var. pubilimba, of which the pericarp thickness is usually about 1 mm or even thinner (Ming 2000; Ming and Bartholomew 2007) (Fig. 3B).

Additionally, regarding life form, *C. angustifolia* is mostly a tall tree, with the tallest individuals reaching up to about 20 m in height (Fig. 2A). In contrast, C. sinensis var. pubilimba typically grows as a shrub that is only 1–3(–5) m tall (Fig. 3A).

Based on these significant differences between the two taxa, Ming (1992)’s taxonomic treatment reducing *C. angustifolia* to C. sinensis var. pubilimba was unreasonable. *Camellia angustifolia* should be regarded as a separate species.

## ﻿Taxonomic treatment

### ﻿ Camellia angustifolia

Taxon classificationPlantaeEricalesTheaceae

Hung T. Chang

960E3941-69DF-53E5-A28D-BE75647D8E27

Camellia angustifolia Hung T. Chang in Acta Sci. Nat. Univ. Sunyats. 20 (1): 96 (Tax. Gen. Camellia 119).1981 et 23 (1): 9. 1984; Hung T. Chang & B. Bartholomew, Camellias 147, pl. 48. 1984; Hung T. Chang in Fl. Guangxi 1: 787. 1991; Hung T. Chang, Fl. Reip. Pop. Sin. 49 (3): 129, pl. 31: 2. 1998.
= C. sinensis var. pubilimba auct. non Hung T. Chang: T.L. Ming in Acta Bot. Yunnan. 14 (2): 129. 1992 et in Monogr. Camellia 135. 2000; T.L. Ming & B. Bartholomew, Fl. China 12: 377. 2007, quoad syn. C. angustifolia.

#### Type.

China Guangxi: • Jinxiu, in shade on slope, 1520 m, 16 July 1958, *Y.K. Li 400664* (holotype: SCBI0003466!; isotype: IBK00190495!) (Fig. 1A, B).

#### Description.

Evergreen arbor, 10–20 m tall. ***New branchlets*** glabrous, ***terminal buds*** sparsely to densely pubescent. ***Petioles*** 5–10 mm long, glabrous; ***leaf blades*** lanceolate, oblong to elliptic, 7–13 × 2–5 cm, coriaceous, abaxially yellowish green, glabrous or subglabrous, adaxially dark green, glabrous, shiny, midrib and secondary veins abaxially elevated and adaxially impressed, secondary veins 10–13 pairs, base cuneate, apex acuminate, margin serrulate. ***Flowers*** axillary, solitary or paired, 4–5 cm in diam. ***Pedicels*** 6–12 mm long, glabrous. ***Bracteoles*** 2 (–3), caducous. ***Sepals*** 5, persistent, suborbicular, 5–9 × 5–10 mm, abaxially glabrous, adaxially sericeous, margin ciliolate. ***Petals*** 7–8 in 1–2 whorls, white, elliptic to obovate, 15–30 × 15–20 mm, glabrous on both surfaces, apex obtuse to rounded, inner 4–5 petals basally connate to each other and adnate to the filament tube for 3–4 mm. ***Stamens*** numerous, 18–22 mm long, in 2–3 whorl; ***filaments*** glabrous, filaments of the outer whorls basally connate for 3–5 mm, filaments of the innermost whorl free. ***Ovary*** 3-loculed, globose to ovoid, densely tomentum. ***Styles*** 1, 16–20 mm long, pubescent almost the entire length, apically 3-lobed for 3–5 mm. ***Capsule*** globose, 3–4 cm in diam., 3-loculed with 1–2 seeds per locule; pericarp 2–3 mm thick. ***Seeds*** fuscous, globose, ca. 1.5 cm in diam., glabrous. Figs 1A, B, 2.

#### Phenology.

Flowering September–October, fruiting August–September.

#### Distribution and habitat.

**Figure 4.**
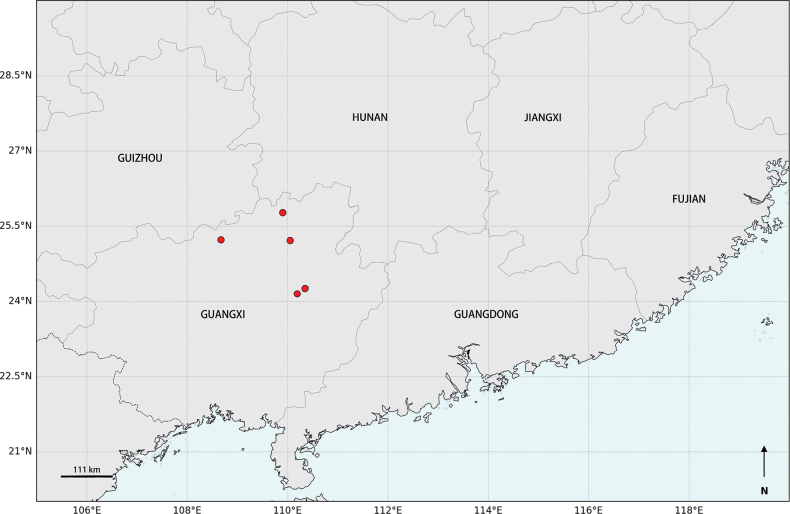
The distribution of *Camellia angustifolia* Hung T. Chang (made by Hanning Duan).

Endemic to northern Guangxi, China (Fig. 4), in the evergreen broadleaf forest at the elevations of 930–1390 m.

#### Additional specimens examined.

**China Guangxi**: • **Jinxiu**, 14 September 2021, *S.X. Yang & P.M. Ye 6752, 6753, 6754, 6755, 6756, 6757* (KUN); 16 February 2023, *S.X. Yang, P.M. Ye et H.M. Tan 7060, 7061, 7062, 7063, 7064, 7065, 7066, 7067, 7068, 7069* (KUN). • **Lingui**, 15 February 2025, *S.X. Yang, Z.S. Liu, Y.L. Pan, H.Q. Deng et H.L. Xiao 7811, 7812, 7813, 7814, 7815, 7816, 7817, 7818, 7819, 7820, 7821, 7822, 7823, 7824* (KUN). • **Longsheng**, 1 May 2025, *S.X. Yang, H.Q. Deng et X.Y. Wang 7941,7942, 7944, 7945* (KUN). • **Rongshui**, 21 July 2014, *X.Q. Yu YXQ125* (KUN); 3 May 2025, *S.X. Yang, Z.S. Liu et H.Q. Deng 7959, 7960, 7961, 7962, 7963* (KUN).

## Supplementary Material

XML Treatment for Camellia angustifolia

## ﻿Additional information

### Use of AI

No use of AI was reported.

### Author ORCIDs

Huiqun Deng https://orcid.org/0009-0004-1622-9923

Xianjun Liao https://orcid.org/0009-0002-3839-022X

Xiangqin Yu https://orcid.org/0000-0001-5245-343X

Zhusheng Liu https://orcid.org/0009-0004-2879-9317

Shixiong Yang https://orcid.org/0000-0001-8472-1275

### Data availability

All of the data that support the findings of this study are available in the main text.
